# Multivariate analysis of immunosenescence data in healthy humans and diverse diseases

**DOI:** 10.3389/fragi.2025.1568034

**Published:** 2025-04-16

**Authors:** Ana Laura Añé-Kourí, Jorge Luis Palomino, Patricia Lorenzo-Luaces, Lizet Sanchez, Nuris Ledon, Karla Pereira, Jenysbel de la Caridad Hernandez, Gisela María Suárez, Beatriz García, Amnely González, Danay Saavedra, Agustin Lage

**Affiliations:** ^1^ Clinical Research Direction, Center of Molecular Immunology, Havana, Cuba; ^2^ Biomedical Sciences Institute, Hasselt University, Hasselt, Belgium; ^3^ Research Direction, Center of Molecular Immunology, Havana, Cuba; ^4^ Laboratory of Immunology, Abu Dhabi Stem Cells Center, Abu Dhabi, United Arab Emirates; ^5^ Diabetes Research Institute, University of Miami, Miami, FL, United States

**Keywords:** immunosenescence markers, multivariate analysis, healthy subjects, centenarians, non-small cell lung cancer

## Abstract

**Introduction:**

Immunosenescence is a dynamic process, where both genetic and environmental factors account for the substantial inter-individual variability. This paper integrates all the data on immunosenescence markers generated in our laboratory and describes the differences and/or similarities between individuals based on their biological conditions (immunosenescence markers) and their associations with chronological age and health status.

**Materials and Methods:**

The dataset consisted of immunological data from healthy donors, centenarians, patients diagnosed with chronic kidney disease, COVID-19 and non-small cell lung cancer (NSCLC), treatment-naïve or treated with platinum-based chemotherapy. To determine whether there are groups of immunologically different individuals despite their age or clinical condition, cluster analysis was performed. Canonical discriminant analysis was performed to determine which variables characterize each cluster.

**Results:**

There are differences in the expression of immunosenescence markers between healthy subjects and patients diagnosed with different pathological conditions, regardless of their age. Meanwhile, the distribution of the clusters indicates the presence of two separate groups of healthy participants, one of them characterized by a high frequency of naïve lymphocytes, and the other with high expression of terminally differentiated lymphocyte subsets. Advanced NSCLC treatment-naïve patients were in the same cluster as a group of healthy subjects. Additionally, centenarians belong to a different cluster than healthy subjects, suggesting they might have a unique immune signature.

**Conclusion:**

The distribution of clusters appears to be more appropriate than univariate associations of single markers for health and disease research. The present work reveals which immune markers are relevant in different physiological and pathological contexts and indicates the need for deeper studies on the biological age of the immune system.

## 1 Introduction

Aging is considered the most important risk factor for most chronic diseases in adulthood, e.g., diabetes, cardiovascular diseases, atherosclerosis, dementia, cancer, etc (Franceschi et al., 2018). However, the aging process is non-linear, as some people remain active and “healthy” while others experience a decline in health or quality of life earlier (Saavedra et al., 2023). The process of aging is highly dependent on the context. Everyone ages differently because each person is unique based on genetics and living history. Even, from an immunological point of view, the combination of the type, intensity, and temporal sequence of antigens to which we are exposed throughout our lives is extremely important in determining the “immunobiography” of the individual (Caruso et al., 2022; Franceschi et al., 2017).

Recent literature highlights the distinction between how old someone is, known as chronological age, and the overall condition of a person’s body, known as biological age. Chronological age is based on a person’s birth, while biological age takes into account several biological, physiological, and environmental factors, such as genetics, diet, and lifestyle (Li et al., 2023). Because individuals of the same age may have different biological ages (Zavala et al., 2024), chronological age is considered an imperfect measure of the aging process because it does not accurately capture an individual’s biological age (Wu et al., 2021). Estimating biological age using a variety of biomarkers, functional assessments, and different models provides valuable insight into an individual’s health status and aging process. By applying these concepts, healthcare providers and researchers can better understand aging, develop more personalized interventions, identify individuals at higher risk for age-related diseases, evaluate the effectiveness of lifestyle interventions or pharmacological treatments for slowing the aging process, and improve health outcomes in different populations and health conditions (Salih et al., 2023).

Aging is also associated with functional changes in immunity, resulting from age-related changes in both the innate and adaptive branches of the immune system. The phenomena that explain these changes have been termed immunosenescence and inflammaging (Saavedra et al., 2023; García Verdecia et al., 2013; Saavedra et al., 2017), which develop over time according to the individual’s immunobiography (Saavedra et al., 2023; Franceschi et al., 2017). Immunosenescence describes the gradual changes in immune function associated with aging, leading to increased susceptibility to infections and age-related inflammatory diseases. This is a highly dynamic and multifactorial process in which some functions decline, while others are maintained or increased due to subject heterogeneity (Saavedra et al., 2023; Caruso et al., 2022; Aiello et al., 2019). Individuals with a history of frequent infections may exhibit a different immune profile compared to those with fewer exposures, impacting their response to infections and vaccines later in life. During the recent COVID-19 pandemic, it was demonstrated that having a “richer” immunobiography (in addition to other factors, including genetic, epigenetic, or metabolic) may adversely affect reactivity to SARS-CoV-2 not only later in life, but also earlier, in young and middle-aged individuals (Witkowski et al., 2022).

Epigenetics, play a pivotal role in regulating aging, age-related diseases, and biological age. These modifications significantly influence both immunosenescence and inflammaging. Recent research has demonstrated that these modifications can alter immune responses and inflammatory pathways, thereby affecting disease outcomes in older populations (Napoli et al., 2023). This highlights the potential of targeting epigenetic factors as therapeutic strategies to address aging-related health challenges and enhance longevity (Costa et al., 2019; Pereira et al., 2024).

In this paper, all the data on immunosenescence markers (lymphocyte immunophenotypes) generated in our laboratory are combined for the first time. Here we describe the differences and/or similarities between individuals based on their biological conditions (immunosenescence markers) and their associations with chronological age and health status.

## 2 Materials and methods

### 2.1 Subjects, data acquisition and processing

The dataset was composed of immunological data from healthy donors, centenarians, patients with chronic kidney disease, COVID-19 patients, treatment-naïve non-small cell lung cancer (NSCLC) patients (who will be named “Before Chemotherapy”), and NSCLC patients who underwent platinum-based chemotherapy (who will be named “After Chemotherapy”).

**FIGURE 1:**
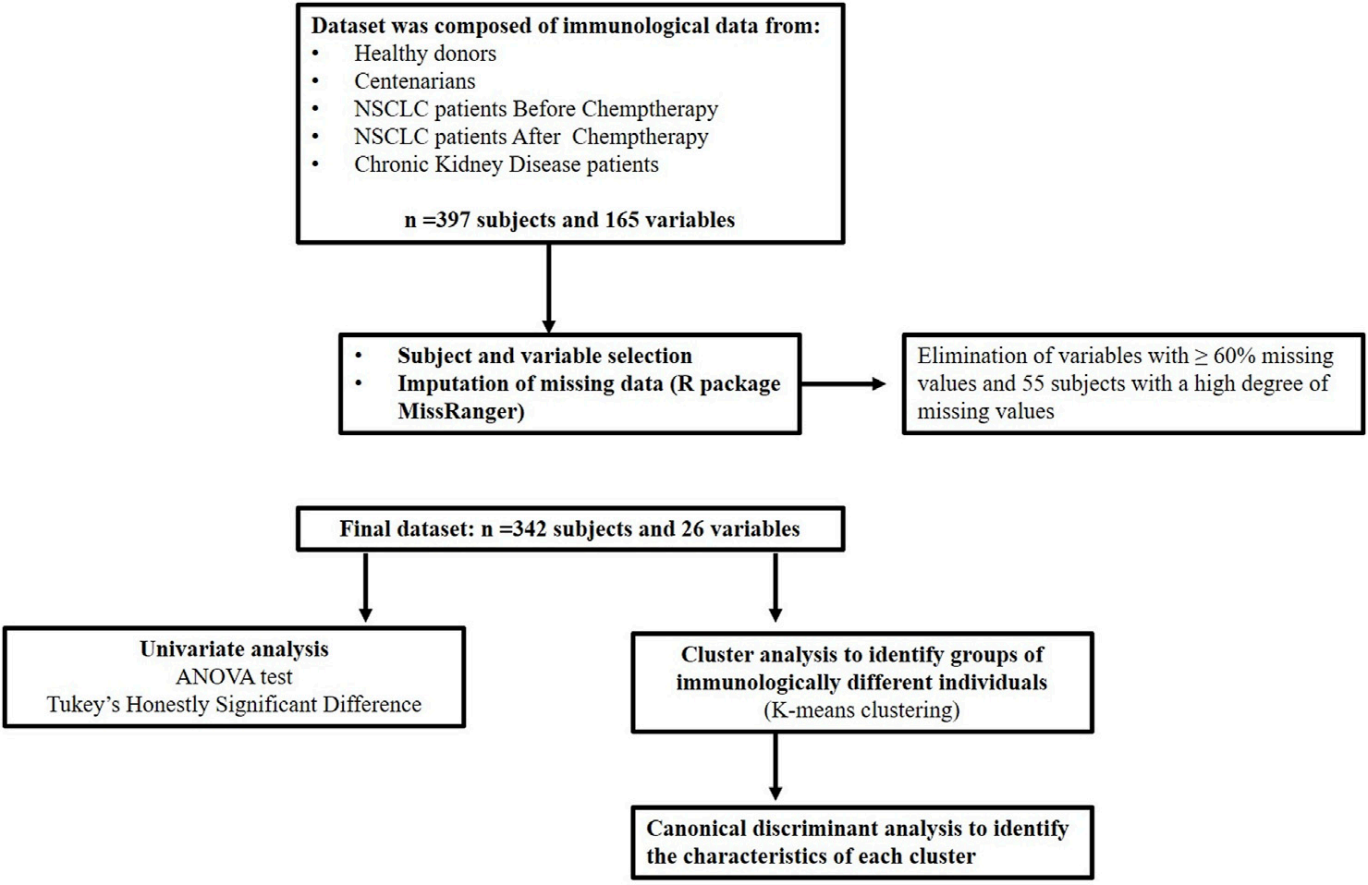
Flow diagram of subject selection, data processing and statistical analysis.

The integration of these data resulted in a dataset of 397 subjects and 165 variables. Subsequently, a variable and subject selection process was performed, followed by imputation of missing data. In this process, variables with more than 60% missing values were eliminated and 55 subjects with a high degree of missing values were discarded. In addition, the R package MissRanger was used for imputation of missing data (Alpert et al., 2019). Imputation of missing data was performed within each subgroup of patients according to their clinical condition. A total of 342 patients and 26 variables were retained as a result (Figure 1).

The distribution of patients according to their clinical condition and the mean of age in each group are indicated in Table 1. All patients are in a similar age range except centenarians. In the case of patients diagnosed with chronic kidney disease, the age of each individual was not available, only the age range of the inclusion criteria of said study.

**TABLE 1:** Distribution of subjects according to their clinical condition and the mean of age.

Clinical condition	n = 342	Age Mean; SD
Healthy subjects	175	62.3 (20.8)
0–39 years old	40	
40–80 years old	135	
Centenarians	43	102.7 (1.86)
Treatment-naive cancer patients	44	60.6 (9.5)
Chemotherapy treated cancer patients	30	65.0 (7.0)
COVID-19 patients	30	58.3 (14.3)
Chronic kidney disease patients	20	NA*

*The age was not available in chronic kidney disease patient’s data.

For the univariate analysis we aimed to compare the immune parameters between healthy subjects and patients with different pathological conditions. Thus, healthy subjects’ stratification was made to achieve an age group similar to those of the patients (between 40 and 80 years old). To determine the influence of age over these changes in the immune system, we then selected only subjects over 60 years old to perform the analysis.

#### 2.1.1 White blood cells collection and staining for flow cytometry

Peripheral blood samples were collected from participants by venipuncture. Red cells were removed from whole blood with lysing solution (NH4Cl, EDTA [tetrasodium], KHCO3). White blood cells were washed twice with cytometry solution (PBS, BSA, azide 20%). Specific antibodies against CD3 (RPE-Cy5, Bio-Rad), CD4 (FITC, BD Pharmingen), CD8 (PE-Cy™7, BD Pharmingen), CCR7 (Alexa-fluor 647, BD Pharmingen), CD45RA (PE-CF594, BD Horizon), CD28 (PE, BD Pharmingen), CD19 (PerCP-Cy5.5, BD Pharmingen), CD27 (PE, BD Pharmingen) and IgD (FITC, BD Pharmingen) were used for staining. Antibodies were initially titrated to determine the optimal conditions for flow cytometry analysis before staining. For surface staining, white blood cells were incubated with the antibody in the dark at 4°C for 20 min. Subsequently, cells were washed twice. Data acquisition was performed with a Gallios Flow Cytometer (Beckman Coulter, 3-laser configuration). The data were processed with FlowJo software (Tree Star Inc., v10[2]), and data exported as tabulated results for statistical analyses. All the data generated were obtained from fresh samples.

The main lymphocyte subpopulations analyzed in the T cell compartment included the stages of differentiation of T (CD3+) lymphocytes: (CD45RA+CCR7+) naive, (CD45RA−CCR7+) central memory, (CD45RA−CCR7−) effector memory, and (CD45RA+CCR7−) effector memory re-expressing CD45RA (EMRA) CD4+ and CD8+ T cells. Additionally, the terminally differentiated subsets CD45RA+CD28−, CD28−, within CD4+ and CD8+ T lymphocytes were evaluated.

As for B cells, the stages of differentiation based on the expression of CD27 and IgD were evaluated: (IgD+CD27−) naïve, (IgD+CD27+) unswitched memory B cells, (IgD−CD27+) switched memory B cells, and (IgD−CD27−) double‐negative B cells.

### 2.2 Statistical analysis

We conducted ANOVA to test differences of the frequency of lymphocyte subpopulations among clinical conditions groups of patients and results was showed in an error bar plot. We performed Tukey’s Honestly Significant Difference (HSD) to adjust for multiple comparisons. Indicating a statistically significant difference was a p value less than 0.05. Package “rstatix” de R was used.

To determine whether there are groups of immunologically different individuals despite their clinical condition, K-means clustering was performed using R (version 4.2.2) with the base ‘kmeans’ function, and the resulting clusters were visualized with 2-dimensional cluster plots using the “factoextra” R package. To determine the optimal number of clusters, we utilized the R package NbClust (version 3.0.1), which offers 30 different indexes based on the methodology proposed by Charrad et al. (2014). The selection of the best number of clusters was conducted according to the majority rule (Charrad et al., 2014).

Neither age nor clinical condition were considered for the cluster analysis, only variables related to their immune status were taken into account.

We used a canonical discriminant analysis (“candisc” package in R) with group centroids, 95% confidence interval, and vectors representing immune related variables, to identify the characteristics of each obtained cluster. It was decided to set statistical significances for only terms with false discovery rate (FDR) < 0.05. The R package ggplot2 was used to display the enrichment findings.

## 3 Results

### 3.1 Immunosenescence markers differ between health and disease

**FIGURE 2:**
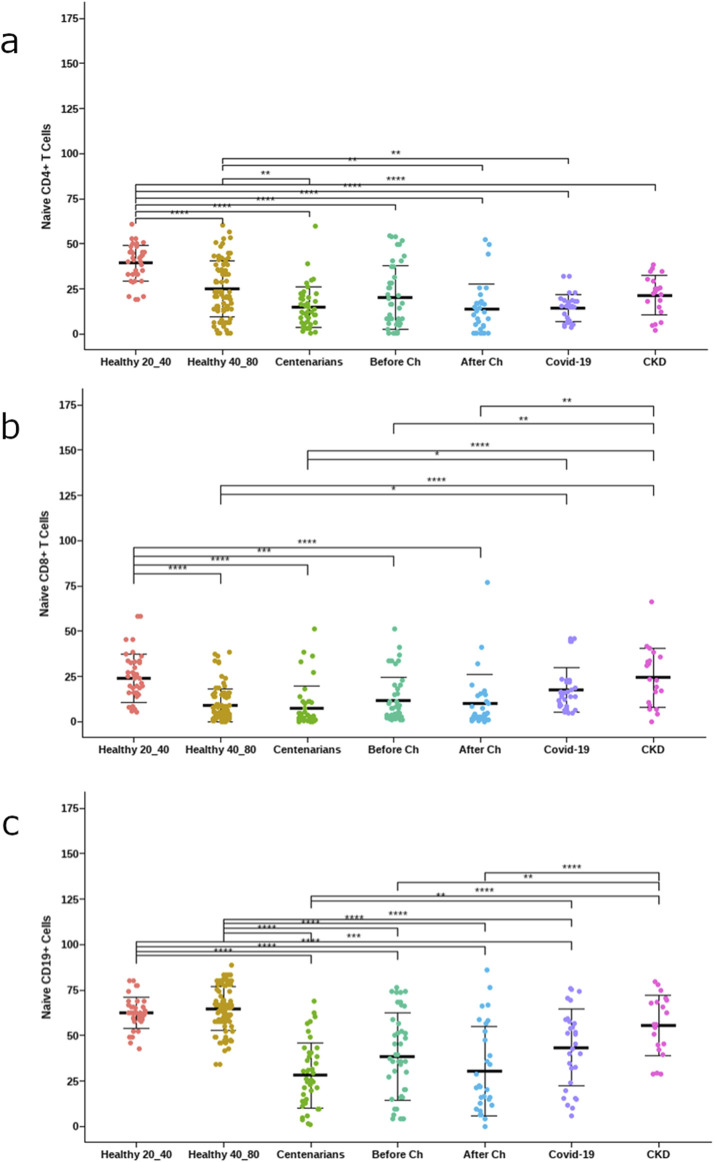
Frequency of naïve lymphocytes. **(a)** CD4 T cells (CD4+CD45RA+CCR7+), **(b)** CD8 T cells (CD4+CD45RA+CCR7+) **(c)** B cells (CD19+CD27−IgD+). The asterisks indicate statistically significant differences among the groups (*p < 0.05) using ANOVA test.

Many differences were observed comparing the frequency of lymphocyte subpopulations among groups. As expected, Figure 2 shows that healthy subjects under 40 years old had the highest frequency of naïve lymphocytes.

**FIGURE 3:**
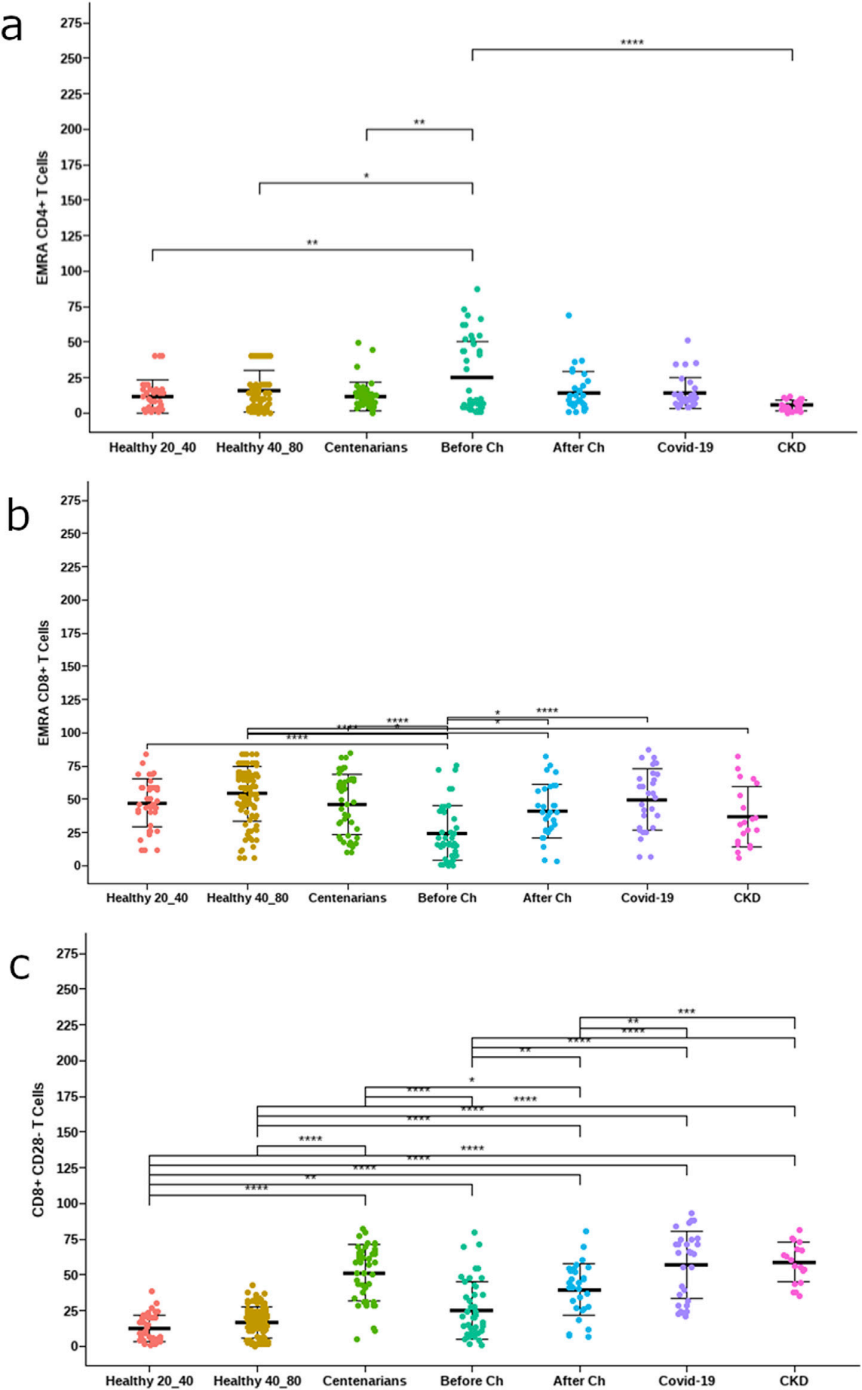
Frequency of late-stage differentiated lymphocytes. **(a)** EMRA CD4 T cells, **(b)** EMRA CD8 T cells **(c)** CD8+CD28− T cells. The asterisks indicate statistically significant differences among the groups (*p < 0.05) using ANOVA test.

Additionally, COVID-19 and cancer patients who underwent chemotherapy displayed significantly lower frequencies of CD4+ naïve T cells compared to healthy donors of the same age range (between the ages of 40–80). Moreover, COVID-19 and cancer patients had significantly lower frequencies of naïve B cells compared to healthy donors in the same age range (Figure 3).

Centenarians, COVID-19 patients, and cancer patients after treatment with platinum-based chemotherapy showed significantly elevated frequencies of CD8+CD28− T cells in comparison to healthy individuals between the ages of 40 and 80. However, the frequencies of EMRA CD8^+^ T cells were lower in treatment-naïve cancer patients (Figure 3).

### 3.2 Subjects with same age and similar clinical conditions were immunologically different

Based solely on the immune phenotype, four clusters were identified as shown in Figure 3. After the cluster analysis, we examined the composition of the clusters according to the clinical condition of the patients.

The first cluster was mainly composed of healthy individuals (median age 69.7 years). The second cluster comprised healthy subjects (median age 57 years) and treatment-naïve NSCLC patients. The third cluster consisted of centenarians and patients with chronic kidney disease. The fourth cluster resulted in a combination of cancer patients (both before and after chemotherapy treatment), COVID-19 patients, and some centenarians (Table 2).

**TABLE 2:** Distribution of subjects in each cluster according to their clinical condition.

Clinical condition	Cluster 1	Cluster 2	Cluster 3	Cluster 4
Centenarians	0	5	18	20
Covid-19	0	4	0	26
After Ch	1	5	8	16
Before Ch	3	18	6	17
CKD	0	1	19	0
Healthy	86	85	0	4

**FIGURE 4:**
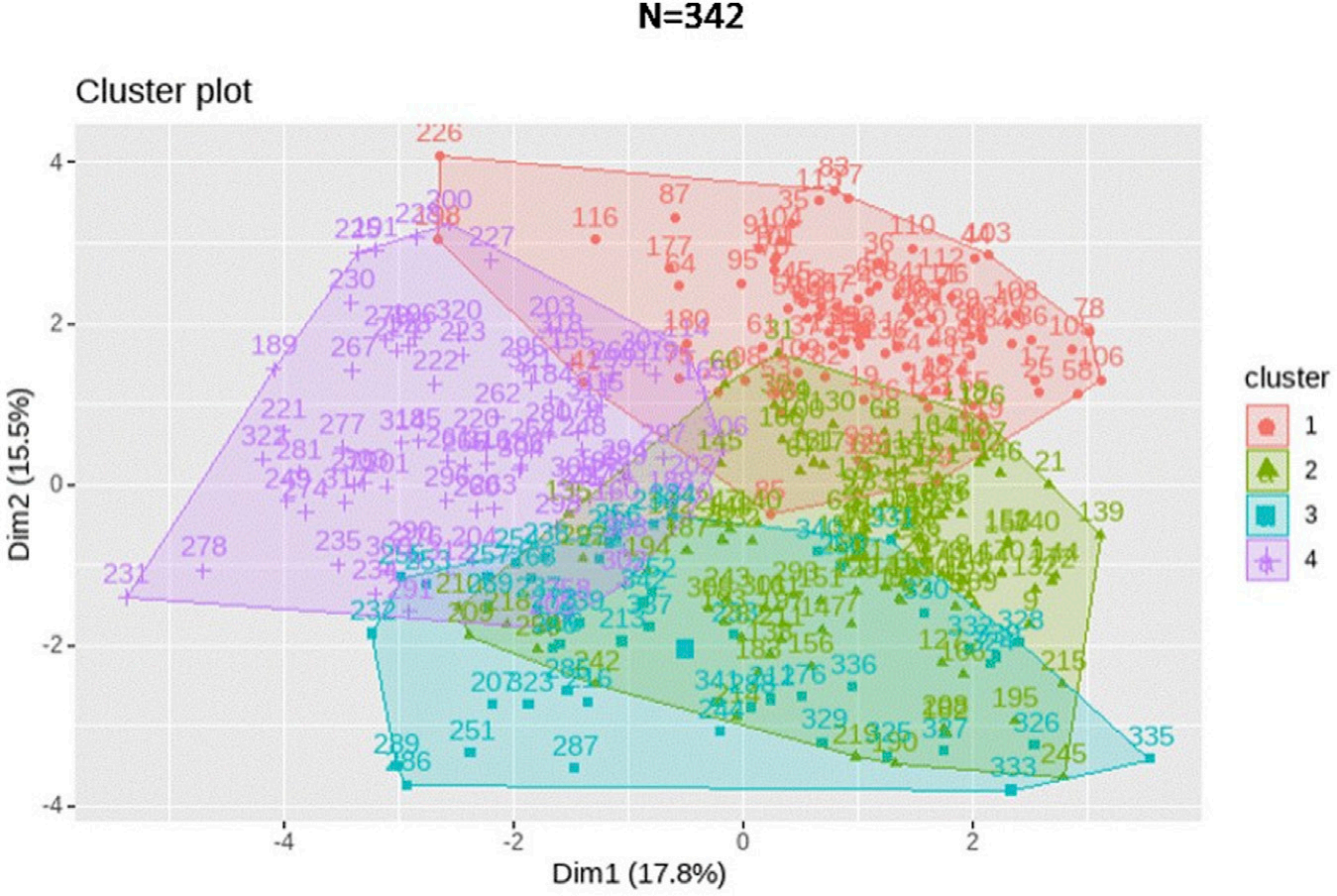
K-means clustering performed for the 397 subjects represented as a 2-dimensional cluster plot based on a principal component analysis.

Notably, the distribution of the clusters showed in Figure 4 and Table 2 indicates the presence of two separate groups of healthy participants, with 86 individuals belonging to cluster 1 and 85 to cluster 2.

Likewise, the centenarians and NSCLC patients prior to undergoing chemotherapy were also segregated into two distinct clusters each.

**FIGURE 5:**
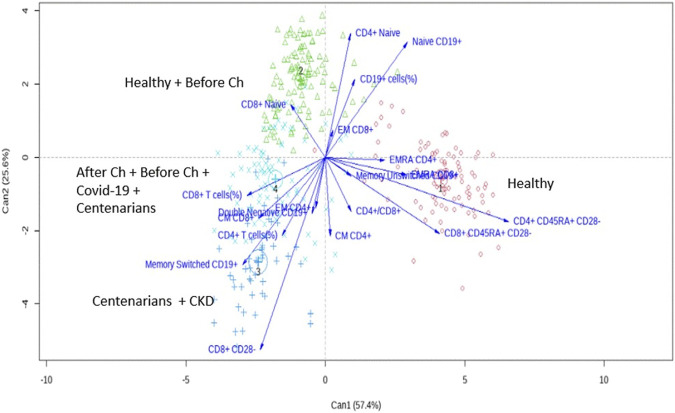
Canonical discriminant analysis biplot with Can1 and Can2 (the 2 firsts canonical dimensions) shows the correspondence of immunological profiles with each four clusters (showed in different colors). Points represent individuals. Vectors represent the correlations of immunological variables with the canonical dimensions.

Then, a discriminant analysis was conducted to identify variables characterizing each cluster. As shown in Figure 5, healthy donors in cluster 1 were characterized by a high expression of the terminally differentiated subpopulations CD4+ CD45RA+CD28− and CD8+ CD45RACD28− T cells. In contrast, cluster 2, which encompassed the other subgroup of healthy subjects, showed a high frequency of CD4+, CD8+ and CD19+ naïve lymphocytes.

Furthermore, centenarians and chronic kidney disease patients assigned to cluster 3, showed a high frequency of CD8+CD28− T cells as well as memory switched CD19+ cells. Interestingly, cluster 4 shows only discrete expression of CD8 T cells (Figure 5).

**FIGURE 6:**
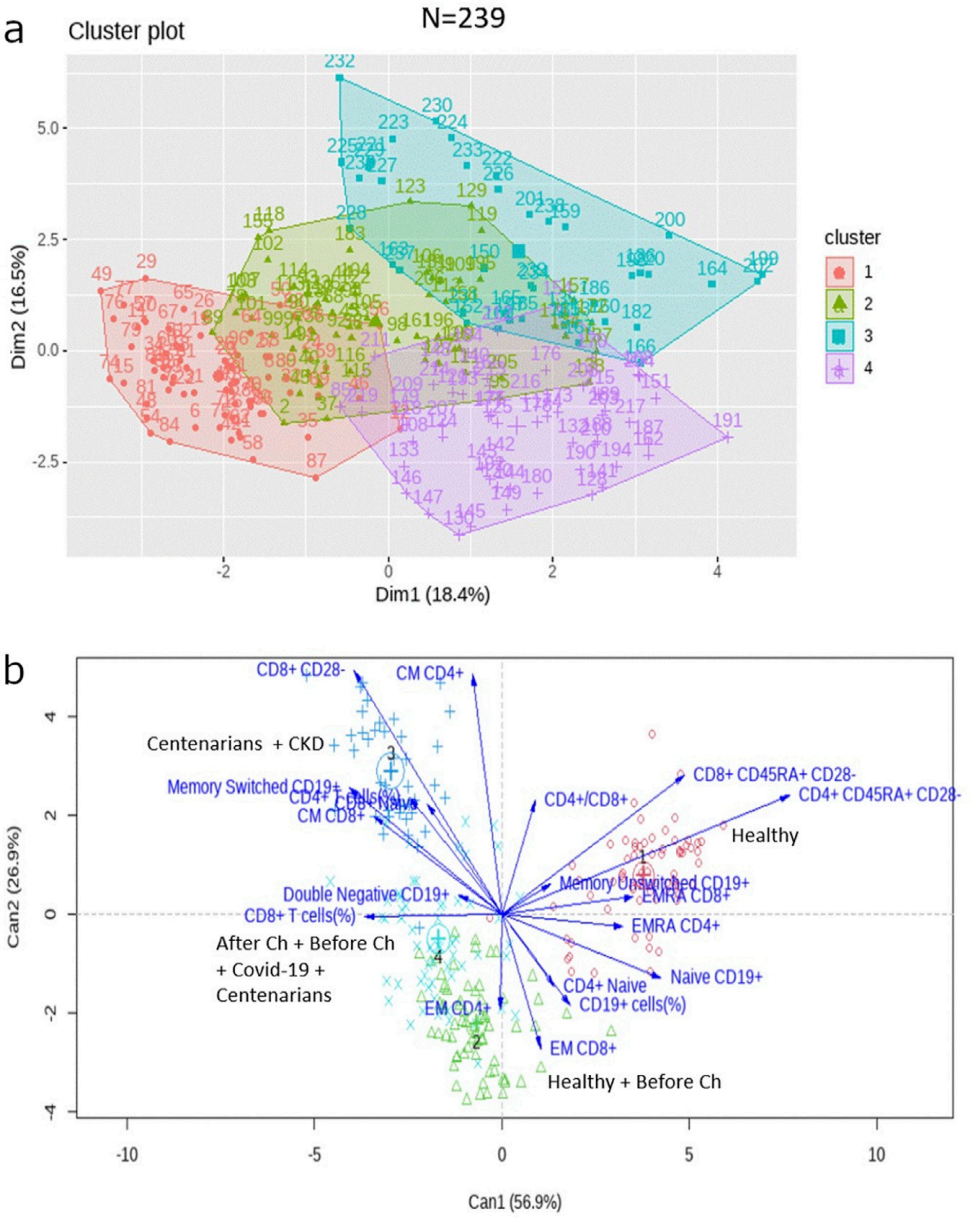
**(a)** K-means clustering performed for the 239 subjects over 60 years old. **(b)** Canonical discriminant analysis biplot for the immunological profiles of the four clusters in patients over 60 years old.

Once again, for subjects over 60 years old, four clusters with a similar distribution were identified (Figure 6a; Table 3) and the same variables were responsible for the separation of the clusters (Figure 6b). The main difference in this case was that naïve cells were no longer represented in the discriminant analysis.

**TABLE 3:** Distribution of subjects over 60 years old in each cluster according to their clinical condition.

Clinical condition	Cluster 1	Cluster 2	Cluster 3	Cluster 4
Centenarians	0	6	16	21
Covid-19	0	1	0	13
After Chemotherapy	0	4	5	13
Before Chemotherapy	0	11	2	9
CKD	0	1	19	0
Healthy	71	45	0	2

## 4 Discussion

Aging is associated with changes in the immune system. We (García Verdecia et al., 2013; Saavedra et al., 2017) and others (Lazuardi et al., 2005; Lioulios et al., 2021; Soto-Heredero et al., 2023) have demonstrated an increase in memory cells and a decrease in naive cells. This gradual change of the immune system with aging has been termed “immunosenescence” (Saavedra et al., 2023). Aging and immunosenescence have been considered as highly relevant risk factors for the development of health-threatening diseases and conditions such as cardiovascular disease, metabolic syndrome, and cancer, among others (Franceschi et al., 2018). However, their relationship is probably much more complex because everyone ages differently, and each individual is unique in terms of genetics and immunobiography (Saavedra et al., 2023; Caruso et al., 2022). Another important reason is that these processes share basic mechanisms (Franceschi et al., 2018).

This work combines for the first time all the data on immunosenescence markers obtained in our laboratory. Presented in an effort to understand the differences and/or similarities among individuals because of their chronological and biological age, and based on their health condition.

We demonstrate here that there are differences in the expression of immunosenescence markers between healthy subjects and patients diagnosed with different pathological conditions, regardless of their age.

As expected, the frequency of naïve T cells was higher in healthy younger subjects and decreased with age and in pathological conditions. As mentioned before, the decline in naïve lymphocytes is a main feature of immunosenescence (Zhang et al., 2021; Bektas et al., 2017). Moreover, the lower frequencies of naïve CD4+ T cells and B cells in advanced NSCLC patients and COVID-19 patients compared with age-matched healthy volunteers is consistent with previous findings (Saavedra et al., 2017). Our group has previously reported low naïve as well as total lymphocyte counts in moderately and severely ill COVID-19 patients and in advanced NSCLC patients treated with platinum-based chemotherapy (Saavedra et al., 2022; Añé-Kourí et al., 2023).

On the other hand, we found that centenarians, COVID-19 patients, and cancer patients after treatment with platinum-based chemotherapy showed elevated frequencies of the terminally differentiated CD28− subpopulation within CD8+ T cells, compared to healthy individuals of similar age range. Strikingly, treatment-naïve NSCLC patients had low frequencies of late-differentiated T cells, which could suggest that the neoplastic disease by itself did not induce pronounced changes in the immune system in this series of data.

Although many differences are observed by univariate analysis, a more comprehensive approach is needed to gain novel insights and understand such a complex system. In this sense, multivariate analysis could be a useful tool to assess the changes in immune profiles with age and disease (Saavedra et al., 2023; Alpert et al., 2019). The main aim of the present work was to explore if there were immunologically different subjects, despite their age or clinical condition. By removing these variables when performing the cluster analysis, we could achieve an unbiased classification of subjects based only on their immune profile.

In this sense, four distinct clusters of subjects were identified. Interestingly, the distribution of the clusters indicates the presence of two separate groups of healthy participants. Furthermore, discriminant analysis shows that the immune profiles of the two groups of healthy subjects differ in the pattern of lymphocyte differentiation; while one of them is characterized by a high frequency of naïve lymphocytes, the other shows high expression of terminally differentiated lymphocyte subsets, classically regarded as immunosenescence markers. We hypothesize that the fact that our model was able to classify healthy subjects into two groups without considering their chronological age, based solely on their expression patterns of immune markers, may be explained by the concept of biological age. While chronological age is based on the date of birth, biological age is a functional measure, and therefore accounts for inter-individual variability. Biological age is able to capture physiological deterioration better than chronological age and is actionable to interventions. As a result, two individuals with the same chronological age could have different biological ages (Chen et al., 2023).

Notably, advanced NSCLC treatment-naïve patients, were in the same cluster as the healthy subjects. This is in line with the previously discussed idea that platinum-based chemotherapy (and not cancer) is a main driving factor of immunosenescence in NSCLC patients (Saavedra et al., 2017; Suárez et al., 2021). Centenarians, on the other hand, were assigned to clusters 3 and 4. Interestingly, they belong to different cluster than the healthy subjects, which suggests that centenarian’s immune signature differs from the rest of healthy subjects. Centenarians were assigned to a cluster where the prevailing marker was a terminally differentiated population (CD8+CD28−), Our previous work on centenarians showed that terminally differentiated populations were predominant in this group of subjects with extreme longevity (Añé-Kourí et al., 2023).

Patients with end-stage renal disease undergoing hemodialysis were a homogeneous group, with the majority of patients assigned to cluster 3. This cluster was mainly associated with the presence of the terminally differentiated population CD8+CD28−. In line with our findings, Crèpin and colleagues described a decrease in naïve CD4+ and CD8+ T cells and an increase in CD28−CD57− terminally differentiated CD4+ and CD8+ T cells when comparing patients with chronic kidney disease (stage IV and dialyzed) and patients without uremia (Crépin et al., 2020).

To the best of our knowledge, there are few studies covering a wide range of immunological parameters in multiple pathological conditions. However, several studies use the advantages of multivariate analysis. In a similar study in a different context, Shapiro and colleagues applied hierarchical clustering analysis after measuring several immune subpopulations in type 1 diabetes (T1D) patients, unaffected controls, and unaffected first-degree relatives of individuals with T1D. They constructed an immune age prediction model in unaffected participants and observed accelerated immune aging in T1D (Shapiro et al., 2023). A study by Granic et al. aimed to explore the associations between immunosenescence profiles and multimorbidity in older adults. Based on clinical information, three different multimorbidity patterns were identified by clustering. However, in this cohort, having a more immunosenescent phenotype, characterized by higher frequency of CD4 and CD8 senescence-like effector memory cells and lower CD4/CD8 ratio, was not associated with multimorbidity (Granic et al., 2022).

Although further longitudinal studies are needed to confirm the results of the present work, there are some elements that emerge as interesting findings. We confirm that (1) univariate analysis, while very important, is not powerful enough to go beyond chronological age measurement to estimate biological age with certainty (2), we need multivariate analysis of data, and cluster identification. A variety of methods for biological age estimation have been developed recently, such as biomarker-based (e.g., epigenetic clocks, telomere length, transcriptomic, proteomic and metabolomics-based predictors) or clinical-based indicators (e.g., frailty phenotype, frailty index, and functional aging). Nevertheless, currently, there is no gold standard for measuring biological age (Chen et al., 2023; Ashiqur et al., 2021; Diebel and Rockwood, 2021).

In the recent scientific literature, many cellular and molecular markers have been associated with age (López-Otín et al., 2023). However, immunosenescence is a complex process, which means that its dynamics cannot be described neither understood by studying their relationship with healthy aging and disease one by one, because their biological impact is rooted in the network of interactions of diverse cells and molecules among themselves. A complex system approach is needed, and a first step in that approach could be the identification of multivariate clusters of data, and the study of the association of each cluster with age and disease (Cohen et al., 2022). Such an approach could, on the one hand, identify different paths towards immunosenescence, and help building a kind of “immunological clock” probably more informative than chronological age itself. On the other hand, the fact that we are able to detect natural groupings of subjects with similar immune profiles, could help discovering predictive biomarkers of healthy versus pathological aging. Additionally, this could be valuable in identifying subjects at risk and provide personalized intervention strategies tailored to the specific needs of each group.

Multivariate analysis and distribution of clusters promises to be more appropriate for health and disease research than univariate associations of single markers because it considers multiple variables simultaneously and is able to capture complex relationships and interactions that univariate methods might overlook (Liu et al., 2023). This is particularly useful when analyzing real-word data, where variables often influence each other. The present work reveals which immune markers are of relevance in different physiological and pathological contexts and indicates the need for deeper studies on the biological age of the immune system. Such studies in Cuban population are currently ongoing.

## Data Availability

The raw data supporting the conclusions of this article will be made available by the authors, without undue reservation.
